# Chemical Profile and In Vitro Protective Effects of *Minthostachys verticillata* (Griseb.) Epling Aqueous Extract in Intestinal Inflammatory Environments

**DOI:** 10.3390/plants15010069

**Published:** 2025-12-25

**Authors:** Angeles Gloria Rodríguez-Basso, Héctor Juan Prado, María Cristina Matulewicz, Karen Perelmuter, Romina Pagotto, Hernán Bach, Susana Beatriz Gorzalczany, Mariela Bollati-Fogolín

**Affiliations:** 1 Universidad de Buenos Aires, Facultad de Farmacia y Bioquímica, Cátedra de Farmacología, Junín 956, Buenos Aires C1113AAD, Argentina; angelesrbasso@gmail.com (A.G.R.-B.); sgorza@ffyb.uba.ar (S.B.G.); 2 Universidad de Buenos Aires, Instituto de Tecnología Farmacéutica y Biofarmacia (InTecFyB), Junín 956, Buenos Aires C1113AAD, Argentina; 3 Consejo Nacional de Investigaciones Científicas y Técnicas (CONICET), Godoy Cruz 2290, Buenos Aires C1425FQB, Argentina; 4 Universidad de Buenos Aires, Facultad de Farmacia y Bioquímica, Cátedra de Tecnología Farmacéutica II, Junín 956, Buenos Aires C1113AAD, Argentina; 5 Universidad de Buenos Aires, Facultad de Agronomía, Cátedra de Química de Biomoléculas, CIHIDECAR-CONICET-UBA, Av. San Martín 4453, Buenos Aires C1417DSE, Argentina; krysia46@gmail.com; 6 Cell Biology Unit, Institut Pasteur de Montevideo, Mataojo 2020, Montevideo 11400, Uruguay; kperelmuter@pasteur.edu.uy (K.P.); pagotto@pasteur.edu.uy (R.P.); mbollati@pasteur.edu.uy (M.B.-F.); 7 Universidad de Buenos Aires, Facultad de Farmacia y Bioquímica, Cátedra de Farmacobotánica y Museo de Farmacobotánica, Junín 956, Buenos Aires C1113AAD, Argentina; hbach@ffyb.uba.ar

**Keywords:** *Minthostachys verticillata*, phytochemical characterization, polyphenols, intestinal barrier, intestinal inflammation

## Abstract

*Minthostachys verticillata* (Griseb.) Epling, commonly known as peperina, is an aromatic species endemic to Argentina and traditionally used for gastrointestinal ailments. Despite its extensive folkloric use and inclusion in the Argentine Pharmacopoeia, its aqueous extract (the most commonly consumed preparation) has been described in terms of major phytochemical groups, and, currently, no studies have investigated its effects on key intestinal epithelial mechanisms. This plant is also employed in the production of beverages and herbal blends, and its massive consumption highlights the importance of its scientific study. Here, the aqueous extract of *M. verticillata* was characterized by liquid chromatography–tandem mass spectrometry, leading to the identification of fourteen polyphenolic compounds. In intestinal cell models, the extract displayed high IC_50_ values, supporting its safety, and exhibited concentration-dependent bioactivity. In HT-29 cells, it modulated NF-*κ*B activation induced by TNF-*α* and reduced LPS-stimulated IL-8 production. Pretreatment of Caco-2 monolayers prevented the decrease in transepithelial electrical resistance, increased FITC–dextran permeability, and nitric oxide production triggered by an inflammatory cocktail. Additionally, the extract inhibited HT-29 cell migration. These results demonstrate that *M. verticillata* aqueous extract exerts anti-inflammatory, barrier-protective, and anti-migratory effects in vitro, providing novel insights into how its polyphenolic composition may underlie these biological activities, supporting its traditional use and potential applications in intestinal health.

## 1. Introduction

The intestinal barrier prevents the entry of pathogenic microorganisms and toxic luminal substances while regulating, at the same time, the absorption of nutrients, electrolytes, and water from the lumen into circulation. Barrier functions are maintained by a complex multilayer system, which includes an external physical barrier and an inner functional immunological component [1,2]. Intestinal chronic inflammatory disorders, such as inflammatory bowel syndrome (IBS) and inflammatory bowel disease (IBD), impair barrier function, which results in elevated intestinal permeability and increased passage of harmful substances from the lumen [3]. Strong evidence suggests the involvement of the nuclear factor kappa B (NF-*κ*B) in the pathogenesis of IBD, a transcription factor which regulates numerous genes that participate in immunological and inflammatory response pathways. Alterations in NF-*κ*B activation have been consistently reported in inflamed colonic tissue of patients with IBD, and several genetic factors contribute to this dysregulation. Variants that enhance NF-*κ*B signaling include alterations in NF-*κ*B target genes, such as those encoding interleukins (IL) 12 and 23 [4]. Additionally, mutations in NF-*κ*B–stimulating immune receptors, such as nucleotide-binding oligomerization domain-containing protein 2 (NOD2), are strongly associated with Crohn’s disease, leading to impaired microbial recognition and contributing indirectly to the chronic inflammatory cycle [5]. In addition, IBD has been linked to an increased production of lipopolysaccharides (LPS) which in turn could be associated with changes in relative abundance or diversity in gut microbiota (dysbiosis). LPS are bacterial surface glycolipids, produced by Gram-negative bacteria, and have been identified as a promoter of diverse inflammatory pathways implicated in the development of IBD. Their potent inflammatory response is mediated mainly by their interaction with Toll-like receptor 4 (TLR-4), determining cytokine cascades (through NF-*κ*B and mTOR/STAT3 pathways) and caspase activation (through NLRP3 inflammasome) in a way that contributes to altered gut permeability, leading to a self-sustaining cycle of inflammation [6].

IBD patients are at a higher risk of developing gastrointestinal cancers due to chronic inflammation and immunosuppressive treatments. Given that the inflammatory environment in IBD resembles that of cancer, with both conditions sharing key mediators such as cytokines and pathways linked to inflammation and oxidative stress, effective control of these processes is essential to reduce the risk of cancer development. Hence, primary prevention strategies often involve inflammation control, achieved with drugs like 5-aminosalicylic acid (mesalazine). In this context, prevention is critical since the coexistence of IBD and cancer makes their treatments even more challenging [7,8].

Due to the chronic nature of these intestinal diseases, inconsistent treatment outcomes of current pharmacological treatments, and related serious side effects, there is a need to seek alternative treatment options [9] and study their molecular mechanism of action.

Among natural options, plant aqueous extracts such as infusions and decoctions, are widely consumed. They are rich in ubiquitous secondary metabolites, like polyphenols, and their intake has been associated with plenty of health benefits [10]. Polyphenols have been proposed as potential intestinal permeability modulators, although the mechanisms involved are not yet fully elucidated. Their activity seems to be attributed to multiple mechanisms, which may also depend upon the type and amount of compounds considered. The results from in vitro studies have shown the capacity of polyphenols to increase the expression and/or production of numerous tight junction proteins and to reduce the release of several interleukins/cytokines [11].

*Minthostachys verticillata* (Griseb.) Epling (*Mv*) is a medicinal plant belonging to the mint family (Lamiaceae) and is the only species that grows in Argentina. It is commonly known as ‘peperina’; its aroma is similar to mint and it is used mainly in infusion as a digestive, sedative, spasmolytic, antidiarrheic, and antiemetic and also to reduce colics and flatulence. Furthermore, this plant finds application in the food industry for the production of liqueurs, refreshing beverages, and herbal blends (comprising *Ilex paraguariensis* mixed with local herbs, used to prepare ‘mate’, the traditional Argentine infusion). It is also commercially available for medicinal and aromatic purposes across various regions of the country [12,13]. Therefore, the massive consumption of its aqueous extract makes its characterization and study important. In addition, it is worth emphasizing that this particular species has been officially recognized and included in the National Argentine Pharmacopoeia [14].

Despite the extensive traditional use of *Mv* as an aqueous infusion, current scientific evidence is largely restricted to studies on the essential oil [15,16,17,18]. Notably, the composition of the aqueous extract has been described in terms of major phytochemical groups. While Bravi et al. (2025) [19] studied silver nanoparticles synthesized with the aqueous extract as a surface disinfectant, reporting flavonoids and tannins acting as particle reducers and stabilizers, this work was oriented toward technological rather than pharmacological applications. Thus, a more exhaustive phytochemical profile is still needed to support mechanistic studies and advance research on its therapeutic potential. The aqueous extract has been evaluated in animal models closely related to IBD and IBS, describing its overall anti-inflammatory outcomes. These include reductions in pro-inflammatory cytokines such as IL-1β and in inducible enzymes such as COX-2 and iNOS [20]. However, the specific epithelial pathways and molecular targets underlying these effects, including those related to intestinal permeability, remain unknown. This gap underscores the need for mechanistic, cell-based studies focused specifically on the aqueous extract, which is the form traditionally consumed by the population.

Hence, the aim of the present work was to characterize the phytochemical composition of the *Mv* aqueous extract and to investigate its effects on key intestinal epithelial mechanisms, including inflammatory signaling, barrier function, and cell migration, using in vitro intestinal models.

## 2. Results

### 2.1. Phytochemical Profile

The assignment of the main components in the *Mv* extract is presented for the first time in Table 1.

**Figure 1:**
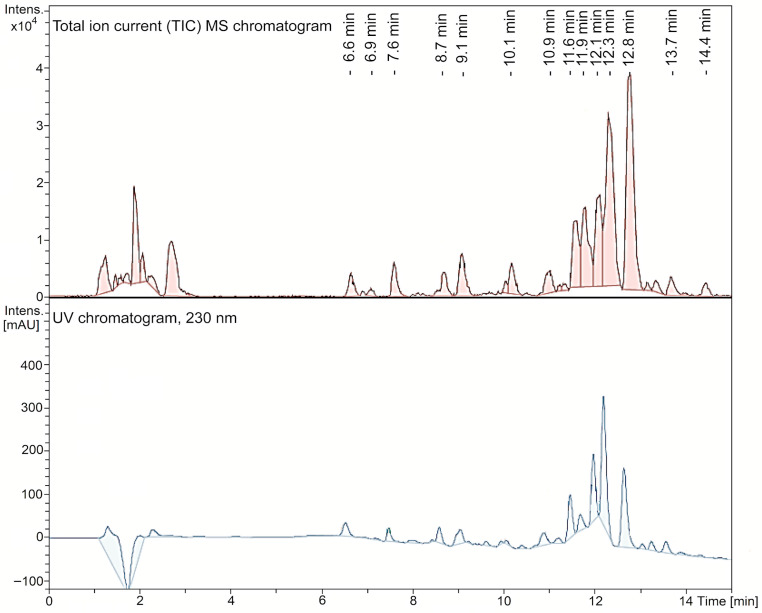
Total ion current mass spectrometry chromatogram and ultraviolet chromatogram at 230 nm of the aqueous *Minthostachys verticillata* extract.

Polyphenols predominate in the aqueous extract. A typical HPLC chromatogram of the aqueous *Mv* extract, obtained with the mass spectrometry detector (total ionic current) and with the ultraviolet detector at 230 nm, is shown in Figure 1. Compounds such as salvianolic acid D, rosmarinic acid, 3-caffeoylquinic acid (chlorogenic acid), 4-caffeoylquinic acid, methyl caffeate dimer, yunnaneic acid F, salvianolic acid B/E isomer 2, isomelitric acid A (caffeoylrosmarinic acid), sagerinic acid, salvianolic acid B, salvianolic acid A and rabdosiin can be considered as caffeic acid derivatives, mostly oligomers. Flavonoids such as rutin and quercetine-3-O-glucoside were additionally found in the extract. Most of these compounds are reported in other species of the genus *Minthostachys* [23] or other members of the Lamiaceae family (Lamiales order), but in none of them in the same combination [21,22,24,28,29,30,31,32,33,34,35,37]. It is important to note that the composition of this aqueous extract was completely different from that of the essential oil of the same species, described in the literature, where the presence of monoterpenes (or monoterpenoids) has been reported, including pulegone (63.4%), menthone (15.9%), and limonene (2.1%), together with other minor components such as α-pinene (0.18%), β-pinene (0.3%), and 1,8-cineole (0.1%), in a gas chromatography analysis [15]. These differences are expected, due to the methods of production, by decoction in this case, and by hydrodistillation in the case of the essential oil.

### 2.2. Cytotoxicity

**Figure 2:**
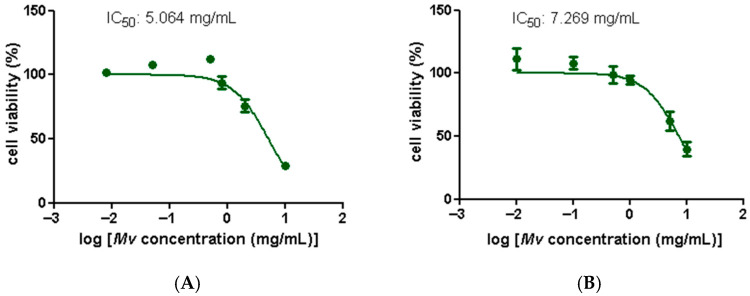
Effect of *M. verticillata* aqueous extract on the cell viability of the intestinal cell lines HT-29 and Caco-2 determined by the MTT assay. (**A**) Cytotoxicity curve in the HT-29 cell line. (**B**) Cytotoxicity curve in the Caco-2 cell line. Dose-response curves with IC_50_ values are shown. Data represent the mean ± SEM of independent experiments. Small error bars are not visible.

Figure 2 displays *Mv* extract cytotoxicity curves for human colon adenocarcinoma cell lines HT29 (A) and Caco-2 (B). *Mv* reduced the number of viable cells in a dose-dependent manner and IC_50_ values were similar in both cell lines. Since high IC_50_ values indicate low cytotoxicity, these results suggest a favorable safety profile for the aqueous extract, which is consistent with its traditional oral use.

### 2.3. NF-κB Pathway Modulation

**Figure 3:**
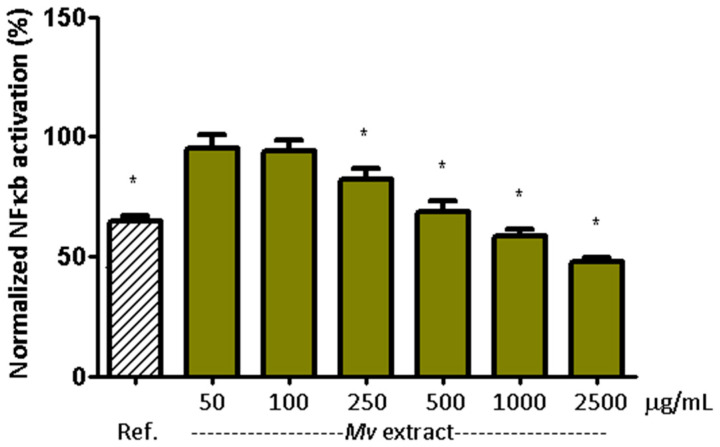
Modulation of TNF-*α*-induced NF-*κ*B signaling in HT-29-NF-*κ*B-hrGFP. Cells were treated with different concentrations of Mv extract for 3 h and 1 ng/mL of TNF-*α* for 24 h. NF-*κ*B activation and cell viability were evaluated by flow cytometry. As a response control, BAY 11-7082 (10 μM) was used. In all cases, cell viability was over 90%. For each treatment group, deviation from the theoretical value (100%) was evaluated using a one-sample Wilcoxon signed-rank test. * *p* < 0.05 compared with 100% as the theoretical value. Ref. corresponds to BAY 11-7082 [38].

**Figure A1:**
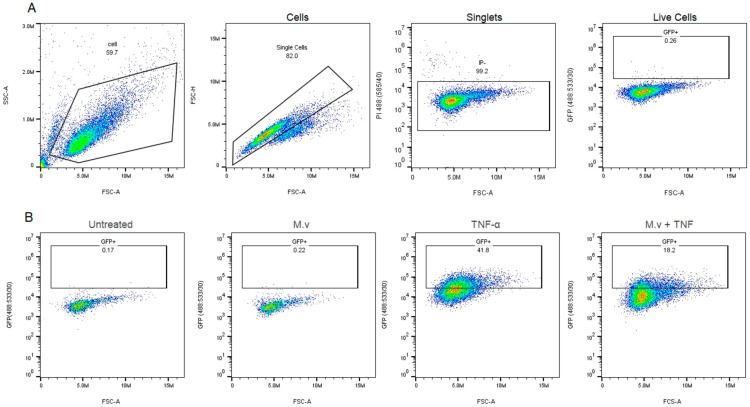
Effect of *M. verticillata* on the HT-29 reporter cell line: NF-*κ*B activation (GFP fluorescence emissions) evaluated by flow cytometry. (**A**) Example of gating strategy: A total of 10,000 events in the singlet gate were recorded. FSC vs. SSC was used to exclude debris. Then, a singlet gate was used to exclude doublets and aggregates (FSC-A vs. FSC-H). A gate for live cells (propidium iodide (PI) negative) was used to exclude dead cells; then, GFP+ cells were determined. (**B**) Representative dot plots of GFP expression in live single cells without treatment, treated with *Mv* (1000 µg/mL), TNF-*α*, or *Mv* (1000 µg/mL) + TNF-*α*.

Figure 3 shows the effect of the *Mv* extract on NF-*κ*B activation in HT-29 reporter cells. The increase in NF-*κ*B signaling induced by TNF-*α* (10 ng/mL) was reduced by the reference inhibitor (BAY 11-7082, 10 µM) [38] and by *Mv* pretreatment in a concentration dependent manner. Values were normalized to the positive (TNF-*α*) and negative (unstimulated) controls. Representative flow cytometry images for NF-*κ*B modulation assays are shown in Appendix A (Figure A1).

### 2.4. IL-8 Production

**Figure 4:**
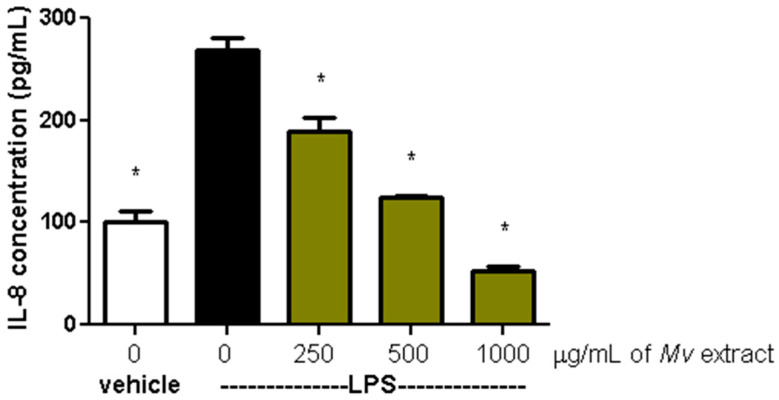
Effect of *Mv* aqueous extract on the production of IL-8 in HT-29 cells induced by LPS. One-way ANOVA analysis (Dunnett’s post-test) compared with LPS control, * *p* < 0.05.

The production of the proinflammatory cytokine IL-8 in HT-29 cells, induced by LPS (100 ng/mL) in the presence or absence of *Mv* extract, is shown in Figure 4. In comparison to cells treated only with LPS, a concentration-dependent reduction in IL-8 release was observed when exposed to the aqueous extract.

Taken together, the findings presented in Section 2.3 and Section 2.4 suggest that *Mv* attenuates the inflammatory response induced by TNF-*α* and LPS.

### 2.5. Intestinal Barrier Functionality

**Figure 5:**
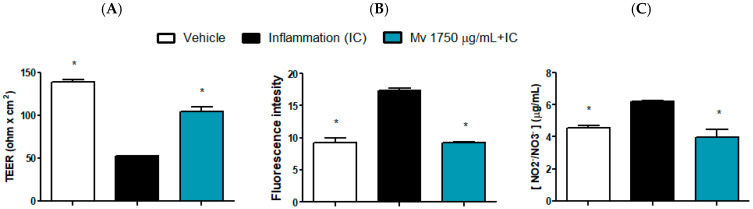
*Mv* effect on intestinal epithelial barrier functionality. TEER (**A**), FITC-D paracellular permeability (**B**) and NO production (**C**). Caco-2 cells were seeded in transwell plates, polarized and challenged with an inflammatory cocktail (IC: TNF-*α* + IL-1β + IFN-ɣ) with or without a 3 h pretreatment with *Mv*. After 24 h, barrier functionality was assessed. Results are expressed as the Mean ± SEM values. One-way ANOVA analysis (Dunnett’s post-test) compared with the IC group, * *p* < 0.05.

Barrier-related endpoints were evaluated using a single, non-cytotoxic concentration of the extract. This concentration was selected to ensure sub-toxic exposure [39] and to enable the integrated assessment of multiple parameters of epithelial function under biologically relevant conditions, rather than focusing on concentration–response characterization for individual markers [40]. Caco-2 and HT-29 models exhibit analogous inflammatory signaling responses [41], supporting the use of either line to investigate barrier and inflammatory endpoints in vitro. Hence, modulatory concentrations observed in HT-29 cells serves as a valid reference to select the concentration for studies in Caco-2 cells. The results in Caco-2 cells on transepithelial electrical resistance (TEER), the passage of FITC-dextran and nitrite/nitrate production in inflammatory condition is shown in Figure 5A–C respectively, where the effect of the *Mv* extract on these parameters can be observed. The inflammatory cocktail (IC) reduced almost 3-fold the normal TEER, nearly duplicated the passage of FITC-dextran and augmented around 50% the production of nitrite/nitrate. These alterations were prevented by pretreatment with *Mv* extract indicating its ability to enhance epithelial barrier functionality, prevent an increase in permeability and display an anti-inflammatory response. The *Mv* extract alone did not show any significant differences from the control group in any of these assays.

### 2.6. In Vitro Cell Migration

**Figure 6:**
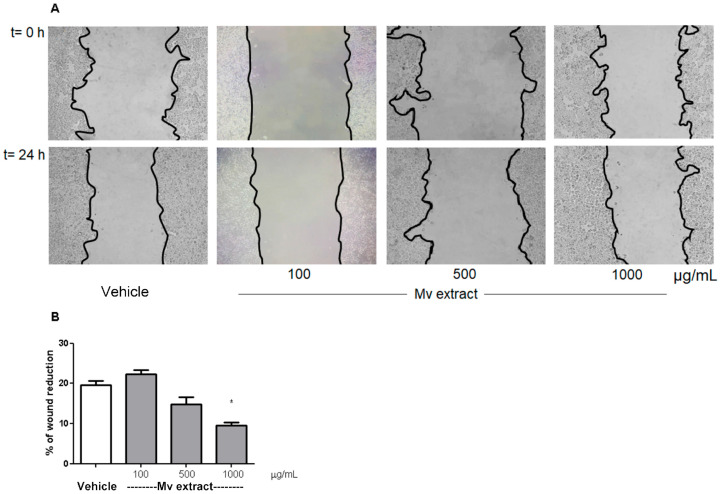
Scratch assay in HT-29 cell line. (**A**) Representative images of wound areas at 0 and 24 h after wounding, following treatment with different concentrations of *Mv* aqueous extract or with fresh medium (vehicle control). (**B**) Quantification of wound closure. One-way ANOVA followed by Dunnett’s post-test compared with vehicle group; * *p* < 0.05.

Figure 6 illustrates the scratch assay results conducted on HT-29 cells. Cell migration was hindered at the highest concentrations of *Mv*, as evidenced by reduced wound area closure when compared to the control cells after a 24-h treatment period.

## 3. Discussion

In recent years, interest in medicinal plants for the treatment of gastrointestinal disorders has increased, not only as sources of new bioactive chemical entities but also as multitarget interventions for complex diseases. In this context, this study provides mechanistic insights that have not been previously described for the biological activity of the traditional *Mv* aqueous extract, which differs in chemical profile and bioactivity from the essential oil that has dominated previous research. Although in vivo studies have demonstrated anti-inflammatory activity of the aqueous extract [20], inhibition of the NF-*κ*B pathway and protection of gut barrier function are identified in this work as key epithelial mechanisms underlying its effects. By characterizing the phytochemical composition of the aqueous extract and linking it to epithelial responses, this work addresses a significant gap in the literature and highlights the biological relevance of the traditionally consumed preparation. The observed effects are relevant considering the central role of these processes in intestinal homeostasis and in the pathogenesis of the increasingly prevalent chronic inflammatory bowel disorders.

The extract reduced NF-*κ*B activation induced by TNF-*α* and IL-8 secretion induced by LPS. LPS and TNF-*α* both activate inflammatory signaling, but through distinct upstream receptors and partially overlapping downstream cascades. LPS binds to TLR4 on the epithelial cell surface, triggering signals that lead to the activation of several transcription factors, including NF-*κ*B. This results in the production of multiple pro-inflammatory mediators, such as IL-8, amplifying mucosal inflammation. On the other hand, TNF-*α* acts through its receptor TNFR, recruiting adaptor proteins which also converge on the activation of the Inhibitor of *κ*B Kinase (IKK) complex and the subsequent nuclear translocation of NF-*κ*B [4]. In intestinal pathophysiology, although the etiology varies, NF-*κ*B signaling represents a convergent point that contributes to chronic inflammation, epithelial barrier disruption, and increased permeability [4,42], supporting the relevance of targeting this pathway. Taking into account both stimuli-induced signaling events, the extract may limit inflammatory amplification and maintain barrier homeostasis. Besides, the ability of the extract to attenuate LPS-responsive pathways is particularly relevant, given their central role in driving mucosal inflammation and barrier disruption in IBD.

On the other hand, the extract also influenced cellular functions related to inflammation-driven processes. Anti-inflammatory responses can affect cell migration by altering the extracellular environment and the signals that cells receive [43], as observed in the scratch assay in HT-29 cells, where the *Mv* aqueous extract prevented migration, a crucial factor in the context of cancer. Since chronic intestinal inflammation is a recognized driver of epithelial transformation and colorectal cancer development [7,8], the ability of the *Mv* aqueous extract to attenuate inflammatory pathways together with limiting migration supports its potential role as a modulator of inflammation-associated tumorigenic mechanisms. Given that cell viability remained above 90% at all tested concentrations, the reduction in wound closure induced by *Mv* could be attributed to modulation of signaling pathways involved in cell migration (e.g., cytoskeleton dynamics, adhesion molecules), rather than cytotoxicity. Several polyphenols present in the *Mv* aqueous extract have reported anti-migratory and anti-invasive potential in diverse tumour cell lines by modulating multiple cellular pathways. Hence, these mechanisms may underlie the reduced wound closure observed in this work. Salvianolic acid B inhibits EMT and stabilizes the cytoskeleton by binding β-actin [44], and also suppresses migration and invasion via the RECK/STAT3 axis [45]. Salvianolic acid A reduces MMP-2 and EMT markers via Raf/MEK/ERK signaling [46]. Rutin attenuated ROS generation and modulating redox-sensitive signaling pathways that govern cytoskeletal remodeling and cell–matrix adhesion [47], and by suppressing MMP-2 and key pathways such as STAT3, Wnt, and PI3K/Akt [48,49]. Rosmarinic acid regulates key migratory pathways, targeting the ADAM-17/EGFR/Akt/GSK-3β axis, likely decreasing MMP-2/MMP-9 expression [50] and activating AMPK [51]. Chlorogenic acids isomers (3- and 4-caffeoylquinic acids) modulates VEGFR2, ERK1/2, and Akt signaling, binds annexin A2, inhibits MMP expression and pro-migratory PI3K/Akt and MAPK pathways, ultimately reducing oxidative stress and extracellular matrix remodeling [52]. Quercetin-3-O-glucoside reduces motility by blocking EGFR signaling [53]. Overall, these findings suggest that the extract’s impact on cell migration likely reflects the combined action of multiple constituents, acting on complementary pathways.

The results in Caco-2 monolayers further reinforce the promising ability of the extract to influence the inflammatory response and intestinal barrier function. Pretreatment with the extract prevented the inflammatory cocktail–induced decrease in TEER and increase in FITC–dextran flux across the monolayers, indicating a potential protective effect on barrier function and suggesting that the extract may strengthen epithelial cohesion and limit permeability increases. Additionally, Caco-2 cells pre-treated with the extract exhibited a decrease in nitrite/nitrate release in the supernatant indicating an anti-inflammatory response. Together, these results suggest that the aqueous extract enhances epithelial cohesion and limits inflammation-induced permeability, two processes highly relevant to the pathophysiology of IBD and IBS.

Although the aqueous extract of *M. verticillata* is the form most widely consumed and traditionally used, its chemical composition has been far less explored than that of the essential oil. This study highlights that the aqueous extract differs markedly from the essential oil [16] and reveals a promising polyphenolic profile with potential relevance for gastrointestinal disorders. In this context, plant extracts from Lamiaceae and other families that share caffeic acid derivatives and flavonoid polyphenols with *Mv* have shown anti-inflammatory effects, improvements in intestinal barrier function and NF-*κ*B inhibition [32,54,55,56,57,58,59]. Other studies have evaluated pure compounds that are also present in *Mv*. In this regard, caffeic acid targets COX-2 and its product prostaglandin E2, as well as the biosynthesis of IL-8 and IL-1β, and it inhibits the formation of advanced glycation end products, exhibiting antioxidant and anti-inflammatory properties in cellular and in vivo contexts [60,61]. Salvianolic acid B shows anti-inflammatory effects and NF-*κ*B regulation [62,63,64]. The main targets of the flavonoid rutin, a prebiotic agent, include inhibition of cyclooxygenase, NF-*κ*B, and Nrf-2 pathways, attenuating oxidative stress and improving barrier function and immunity [65,66,67,68,69]. Therefore, although these data could support the plausibility of the effects observed in the present work, the biological activity of the extract is likely to result from the integrated and potentially synergistic action of its complex mixture of constituents rather than from a single isolated compound, underscoring the importance of studying whole extracts.

Overall, the ability of *Mv* aqueous extract to modulate the NF-*κ*B–related inflammatory signaling, improve intestinal barrier function, and limit migration is highly relevant, as these interconnected epithelial mechanisms play central roles in the pathophysiology of chronic intestinal disorders, where epithelial dysfunction is a key perpetuating factor. This modulatory capacity provides a mechanistic framework that is consistent with its traditional digestive use and, along with the previously reported in vivo effects [20], supports the therapeutic potential of this widely consumed preparation. It further highlights the relevance of its polyphenolic composition in mediating its biological activity on the digestive system and reinforces the importance of exploring whole extracts as complementary strategies. Thus, these results position the extract as a promising candidate for future translational investigations in inflammation and barrier-related intestinal disorders.

## 4. Materials and Methods

### 4.1. Drugs and Materials

Culture media, fetal bovine serum (FBS), and cell culture reagents were provided from Gibco (Thermo Fisher Scientific Inc., Waltham, MA, USA), Life Technologies (Carlsbad, CA, USA), GE Healthcare (Chicago, IL, USA), and Greiner (Kremsmünster, Austria). Plasticware was obtained from Corning Inc. (Corning, NY, USA). IL-1β was purchased from Invitrogen (Thermo Fisher Scientific Inc., Waltham, MA, USA) and lipopolysaccharide (LPS), Griess reagent and TNF-*α* were obtained from Sigma (Merck, Darmstadt, Germany). Unless otherwise indicated, all other chemical reagents employed were of the highest available grade and acquired from Sigma.

### 4.2. Plant Material and Extract Preparation

The aerial parts (leaves, stems and flowers/fruits) of *Minthostachys verticillata* (Griseb.) Epling, ‘peperina’, were collected in Instituto de Recursos Biológicos, Plantas Medicinales y Aromáticas, Hurlingham, Province of Buenos Aires, Argentina and identified by agronomist Hernan Bach. A voucher specimen (H. G. Bach. 738) was stored in Herbario de Farmacobotánica (BAF), Facultad de Farmacia y Bioquímica, Universidad de Buenos Aires, Argentina. The plant material was grounded into a fine powder and a decoction was prepared following the corresponding Farmacopea Argentina 7th ed. monograph [70]. The extract was concentrated, lyophilized and stored at 4 °C (Yield: 38%).

To perform the different assays, stock solutions of the extract were prepared in sterile phosphate buffer saline (PBS) and then filtered through a 0.22 µm membrane. Subsequently, the concentrations of interest were prepared in the culture medium corresponding to each test.

### 4.3. Phytochemical Profiling of the Extract

HPLC-DAD-ESI-MS/MS analyses were performed employing an Agilent 1200 HPLC system (Agilent Technologies, Santa Clara, CA, USA) equipped with a binary pump (model G1312B), an automatic injector (model G1367D), a degasser (model G1379B), and a photodiode array detector (model G1315C) registering the chromatograms at 230 nm and 330 nm. The system was coupled to a high-resolution mass spectrometer Bruker micrOTOF-QII (Bruker Daltonics, Billerica, MA, USA) with an electrospray ionization source (ESI).

A Phenomenex Luna C18 column (150 mm × 2 mm, 3 μm particle size, 100 Å pore size), with a mobile phase of water: formic acid (98:2 *v*/*v*) (A) and methanol: formic acid (98:2 *v*/*v*) (B), a 0.2 mL/min flow rate and a 20 μL injection volume was used in the study. A multistep gradient was used, starting with 10% B at 0 min, 40% B at 2 min, 60% B at 10 min, 75% B at 12 min, 10% B at 12,4 min up to a total running time of 18 min. The ionization conditions were 200 °C and 3.5 kV for the capillary temperature and voltage, respectively. Nitrogen was employed as nebulizer gas, with a pressure of 3.5 bar, and as drying gas with a flow of 7.0 L/min. The mass scan range was 100–1000 *m*/*z* in negative mode. Data was acquired and processed with the Bruker Compass Data Analysis software V. 4.0. The assignment of the main components in the *M. verticillata* extract was achieved by comparison of their retention times and UV spectra with those of authentic standards and by comparison of their mass spectrum and fragmentation with literature.

### 4.4. Cell Lines and Culture Medium

HT-29 NF-*κ*B reporter cell line (HT29-NF-*κ*B-hrGFP) [41] were cultured in RPMI1640 (Life Technologies), HT-29 (ATCC HTB-38) and Caco-2 (ATCC HTB-37) cells were cultured in DMEM glutaMAX™ (Life Technologies, USA). Unless otherwise indicated, all culture media were supplemented with 10% (*v*/*v*) FBS (Life Technologies). Cells were propagated in 25 or 75 cm^2^ tissue culture flasks and incubated at 37 °C in a 5% CO_2_ humidified atmosphere to reach approximately 80% confluence. Afterwards, cells were trypsinized, their concentration was adjusted by manual cell counting with Neubauer chamber and were used for various experimental purposes. Less than twenty cell culture passages were made in all described assays.

### 4.5. Cytotoxicity Assay

Cytotoxicity was determined by the 3-(4,5-dimethyl- thiazole-2-yl)-2,5-diphenyltetrazolium bromide (MTT) colorimetric assay [71]. Briefly, 4 × 10^4^ HT-29 cells or 3 × 10^4^ Caco-2 cells per well were seeded in a 96-well plate and incubated at 37 °C for 24 h. After removing culture supernatants, *Mv* extract (0.01–10.00 mg/mL) in media supplemented with 10% FBS were added and incubated at 37 °C for 24 h. Then, the medium was changed and the cells were incubated with 0.1 mL MTT 0.5 mg/mL under normal culture conditions for 3 h. The resulting formazan precipitate was dissolved in DMSO:isopropanol (1:1, 100 µL per well) and measured at 570 nm with a microtiter plate reader to assess cell viability. Non-linear regression graphs were plotted and the 50% cytotoxic concentration (IC_50_) values were calculated. Every IC_50_ is the average of at least three independent determinations.

All assays were conducted using *Mv* concentrations that did not compromise cell line viability. To ensure the absence of toxicity, concentrations below the IC_50_ were employed.

### 4.6. Modulation of NF-κB Pathway

The modulation effect of the NF-*κ*B pathway on HT29-NF-*κ*B-hrGFP was analyzed [41]. Briefly, cells were seeded in 96-well plates at a density of 3 × 10^4^ cell/well and after 24 h, cells were preincubated for 3 hs with *Mv* at final concentrations of 50, 100, 250, 500, 1000, 2500 µg/mL. Each concentration was assayed in triplicate. Afterwards, TNF-*α* was added (concentration per well: 1 ng/mL), and cells were incubated for 24 h at 37 °C in a 5% CO_2_ humidified atmosphere. At the end of the incubation time, supernatants were collected and stored at −80 °C for later IL-8 quantification. Cells were trypsinized and analyzed by flow cytometry to quantify NF-*κ*B activation. Propidium Iodide (PI) was added to analyze cell viability. A BD Accuri™C6 (BD Bioscience, Franklin Lakes, NJ, USA) flow cytometer was used and BD Accuri™C6 software V1.0.264.21 was used for data acquisition. GFP and PI fluorescence emissions were detected using band-pass filters 533/30 and 585/40, respectively. For each sample, 10,000 counts gated on an FSC versus SSC dot plot, excluding doublets, were recorded. Only single living cells (cells that excluded PI) were considered. Cells without treatment and cells treated only with the stimulus or the different extracts were included in the assay but not for statistical analysis. BAY 11-7082 (10 μM) [38], an inhibitor of the translocation of the transcription factor to the nucleus, was used as a reference drug. Groups included: (i) Vehicle control (unstimulated cells), (ii) TNF-*α* control (positive control for NF-*κ*B activation), (iii) *Mv* aqueous extract, and (iv) BAY 11-7082 (10 µM) as a reference drug for NF-*κ*B inhibition. NF-*κ*B activation was normalized by assigning the fluorescence values of the vehicle control as 0% response and those of the TNF-*α* control as 100% response. At least three independent experiments were performed.

### 4.7. IL-8 Production by LPS

HT-29 cells were cultured in 48-well plates with a seeding density of 6 × 10^4^ cell/well and incubated for 24 hs. Cells were preincubated for 3 hs with *M. verticillata* at final concentrations of 250, 500, 1000 µg/mL. Cells were then activated with LPS (100 ng/mL) for 24 h. At the end of the incubation time supernatants were collected and stored at −80 °C until the IL-8 quantification was performed. At least three independent experiments were carried out. IL-8 was determined by enzyme-linked immunosorbent assay (ELISA) according to the manufacturer’s instructions (Elisa Max™ Deluxe Set Human IL-8, Biolegend, San Diego, CA, USA). Absorbance was read at 450 and 570 nm within 15 min using a Multiskan FC microplate spectrophotometer (Thermo Fisher Scientific Inc.). The absorbance at 570 nm was subtracted from the one at 450 nm. Each concentration was assayed in triplicate within one experiment, and at least three independent experiments were performed.

### 4.8. Transepithelial Electrical Resistance (TEER)

Caco-2 cells were seeded in 12-well transwell plates (polycarbonate membrane with 0.4 µm pore size) at a concentration of 1 × 10^5^ cell/well and incubated for 21 days to achieve cell polarization. The volume of medium maintained on both sides of the transwell was 500 µL and was renewed every 48 h. A TEER value of 200–300 Ω.cm^2^ was considered to prove intact Caco-2 monolayers with well-developed tight junctions.

Four groups were evaluated: normal (untreated control), *Mv* extract control, inflammatory cocktail (IC) control and IC with *Mv* extract (3 hs pretreatment). Each group was analyzed in triplicate per experiment. The concentration of *Mv* per well was 1750 µg/mL, selected based on preliminary MTT assays in Caco-2 cells and corresponding to one-quarter of the IC_50_, thus ensuring a strictly sub-toxic range and minimizing potential interference from cellular stress, as recommended for functional barrier assays [38]. Given that Caco-2 and HT-29 cells share similar responses to common inflammatory stimuli [40], the modulatory concentrations of the NF-*κ*B signaling pathway identified in HT-29 cells was considered a valid reference for Caco-2 barrier assays. Membrane injury was induced in both chambers by IC which was composed of 50 ng/mL of each cytokine (IFN-γ, TNF-*α* and IL-1β) per well. TEER measurements were carried out before any treatment was made (basal) and 24 h after IC was added to the corresponding groups, according to a previous reported protocol [72] with minor modifications. Briefly, culture medium was aspirated and stored at −80 °C for further nitric oxide production measurement, then Caco-2 cell monolayers were rinsed with 500 µL of PBS. A volume of 0.7 mL of PBS was transferred to the apical side and 2.1 mL to the basolateral chamber. Using the MilliCell^®^-ERS volt-ohm meter (Merck Millipore, Burlington, MA, USA) the resistance of an electrical current between electrodes was measured by immersing the electrode at a 90° angle, with the longer tip in the basolateral chamber and the other in the apical one. Caution was required to avoid touching the cell monolayer with the electrode. The resistance value from each well was corrected by the basal and multiplied by the insert membrane area, so that the results were expressed as Ω.cm^2^. Measurements were recorded at RT and three independent repetitions were made. Cells were manipulated in a biosafety hood to maintain sterility.

Upon completion of the test, both sides of the monolayers were washed three times with PBS before continuing with the assay described in the next section.

### 4.9. Fluorescein Isothiocyanate-Dextran (FITC-D) Assay

The Caco-2 polarized monolayers were employed for this experiment following a previously reported protocol [73] with minor modifications to measure the paracellular permeability. Following the three washes with sterile PBS, a volume of 200 µL of a 0.2 mg/mL FITC-D (4000 KDa MW, Sigma) solution prepared in DMEM glutaMAX^TM^ culture medium without phenol red, was transferred to the apical side of each transwell. In the basolateral chamber, 800 µL of the same culture medium was added. Cells were incubated in a humidified incubator for 4 h. Time elapsed, and an aliquot of each transwell was taken from the basal side of each transwell. To make a blank reading, DMEM glutaMAX^TM^ without phenol red was employed. Fluorescence was measured using a Varioskan^TM^ Lux (SkanIt RE 5.0 software, Thermo Fisher Scientific Inc.) fluorometer microplate reader, selecting 490 nm and 520 nm as excitation and emission wavelengths, respectively.

### 4.10. Nitric Oxide (NO) Production Measurement

NO production by nitric oxide synthases (NOS) activity was indirectly determined in the collected cultured Caco-2 (Section 4.8) media using the Griess reaction [74]. This reaction measures NO_2_^-^ ion, a breakdown product of NO. A volume of 50 μL of supernatant was transferred to a 96-well plate, and the same quantity of Griess reagent was added. A calibration curve using NaNO_2_ was built. The reaction was carried out in the dark for 20 min at RT, and absorbance was measured at 562 nm using a Multiskan FC microplate spectrophotometer (Thermo Fisher Scientific Inc.).

### 4.11. Scratch Assay

HT-29 cell lines were tested through the scratch assay [75] to determine the capacity of these cells for wound closure through migration. Cells were seeded at a concentration of 6 × 10^5^ cell/well into 12-well plates for 24 h incubation. The scratch wounds were made using a sterile 0.2 mL pipette tip. Cell monolayers were subsequently rinsed three times with PBS for debris removal, followed by incubation with *Mv* extract (100, 500, 10,000 µg/mL) in medium or fresh medium for 24 h. At least three independent determinations were made. The wound areas were photographed by an Olympus IX81 inverted microscope (Olympus Corporation, Tokyo, Japan) at 0 and 24 h. The effect of samples on wound closure was analyzed using ImageJ software, version 1.53t and the percentage in each group was calculated with respect to time zero. At least three independent experiments were carried out.

### 4.12. Statistical Analysis

The statistical analysis was carried out using the Instant statistical package GraphPad Prism software, V. 5.04 [76] and R software, V. 3.5.1 [77]. Data are presented as the mean ± standard error of the mean (SEM). Statistical significance of differences between groups was assessed by means of analysis of variance (one way ANOVA) followed by Dunnett’s test. For comparisons against a hypothetical value (100%), a one-sample Wilcoxon signed-rank test was applied. *p* values of 0.05 were considered significant. Randomization and blinding of groups were conducted in all the experiments.

## 5. Conclusions

These findings offer an encouraging outlook for understanding the effects of the aqueous extract of *Minthostachys verticillata* on intestinal function and its potential therapeutic relevance. The extract showed a complex polyphenolic profile and exerted anti-inflammatory, barrier-protective, and anti-migratory effects in intestinal cell models, providing a mechanistic basis for its traditional gastrointestinal use. Furthermore, given the growing interest in functional foods, these results highlight its potential as a natural source of intestinal health-promoting agents and provide a basis for future research and product development. However, additional non-clinical and clinical studies will be necessary to confirm and expand the scope of these findings.

## Figures and Tables

**Table 1:** Phytochemical analysis of *Minthostachys verticillata* aqueous extract.

Peak Number	Retention Time (min)	[M-H]- (*m*/*z*)	Proposed Identity	Area of Identified Peaks at 230 nm (%)	Reference
1	6.6	417	Salvianolic acid D	3.1	[21,22]
2	6.9	359	Rosmarinic acid	0.1	[23,24]
3	7.6	353	3-Caffeoylquinic acid (chlorogenic acid)	1.6	[25,26]
4	8.7	353	4-Caffeoylquinic acid	2.6	[25,26]
5	9.1	387	Methyl caffeate dimer	4.2	[27]
6	10.1	597	Yunnaneic acid F	1.3	[28,29]
7	10.9	717	Salvianolic acid B/E isomer 2	3.9	[30,31]
8	11.6	609	Rutin	9.8	[23,25]
9	11.9	463	Quercetine-3-O-glucoside	7.7	[23,25]
10	12.1	537	Isomelitric acid A (caffeoylrosmarinic acid)	18.0	[32,33]
11	12.3	719	Sagerinic acid	29.4	[28,34]
12	12.8	717	Salvianolic acid B	15.7	[24,30]
13	13.7	493	Salvianolic acid A	2.6	[28,30]
14	14.4	717	Rabdosiin	0.1	[35,36]

## Data Availability

The data that support the findings of this study are available from the corresponding author, upon reasonable request.

## Abbreviations

The following abbreviations are used in this manuscript:

ADAM-17	A disintegrin and metalloproteinase domain 17
Akt	Protein Kinase B
AMPK	AMP-activated protein kinase
BAY 11-7082	NF-*κ*B translocation inhibitor
Caco-2	Human colon adenocarcinoma cell line
COX-2	Cyclooxygenase-2
EGFR	Epidermal Growth Factor Receptor
EMT	Epithelial–mesenchymal transition
ERK-1/2	Extracellular signal-Regulated Kinase 1/2
FITC	Fluorescein isothiocyanate
GSK-3β	Glycogen Synthase Kinase 3 beta
HPLC	High-performance liquid chromatography
HT-29	Human colorectal adenocarcinoma cell line
HT29-NF-*κ*B-hrGFP	Human epithelial NF-*κ*B reporter cell line
IBD	Inflammatory bowel disease
IBS	Inflammatory bowel syndrome
IC	Inflammatory cocktail (IFN-γ, TNF-*α* and IL-1β)
IC_50_	Half maximal inhibitory concentration
IFN-γ	Interferon gamma
IKK	Inhibitor of *κ*B Kinase
IL-1β/8/12/23	Interleukin-1β/8/12/23
iNOS	Inducible nitric oxide synthase
LPS	Lipopolysaccharides
MAPK	Mitogen-Activated Protein Kinase
MEK	Mitogen-activated protein kinase/extracellular signal-regulated kinase kinase
MMP-2/9	Matrix metalloproteinases-2/9
Nrf-2	Nuclear factor erythroid-2-related factor 2
mTOR	Mammalian target of rapamycin
*Mv*	*Minthostachys verticillata*
NF-*κ*B	Nuclear factor kappa B
NLRP3	NOD-like receptor pyrin domain 3
NOD-2	Nucleotide-binding oligomerization domain-containing protein 2
PI3K	Phosphoinositide 3-kinase
RAF	Rapidly Accelerated Fibrosarcoma quinase
RECK	Reversion-inducing Cysteine-rich Protein with Kazal Motifs
SEM	Standard error of the mean
STAT3	Signal transducer and activator of transcription 3
TLR-4	Toll–like receptor 4
TNF-*α*	Tumor necrosis factor alpha
TNFR	TNF-*α* receptor
VEGR-2	Vascular Endothelial Growth Factor Receptor-2
Wnt	Wingless/Integrated
